# Exploring a multi-path U-net with probability distribution attention and cascade dilated convolution for precise retinal vessel segmentation in fundus images

**DOI:** 10.1038/s41598-025-98021-z

**Published:** 2025-04-18

**Authors:** Ruihong Zhang, Guosong Jiang

**Affiliations:** School of Computer, Huanggang Normal University, Huanggang, Hubei 438000 China

**Keywords:** Image segmentation, Convolutional neural networks, Diagnosis, Boosting algorithm, Probability distribution attention, Diseases, Health care, Mathematics and computing

## Abstract

While deep learning has become the go-to method for image denoising due to its impressive noise removal Retinal blood vessel segmentation presents several challenges, including limited labeled image data, complex multi-scale vessel structures, and susceptibility to interference from lesion areas. To confront these challenges, this work offers a novel technique that integrates attention mechanisms and a cascaded dilated convolution module (CDCM) within a multi-path U-Net architecture. First, a dual-path U-Net is developed to extract both coarse and fine-grained vessel structures through separate texture and structural branches. A CDCM is integrated to gather multi-scale vessel features, enhancing the model’s ability to extract deep semantic features. Second, a boosting algorithm that incorporates probability distribution attention (PDA) within the upscaling blocks is employed. This approach adjusts the probability distribution, increasing the contribution of shallow information, thereby enhancing segmentation performance in complex backgrounds and reducing the risk of overfitting. Finally, the output from the dual-path U-Net is processed through a feature refinement module. This step further refines the vessel segmentation by integrating and extracting relevant features. Results from experiments on three benchmark datasets, including CHASEDB1, DRIVE, and STARE, demonstrate that the proposed method delivers improved segmentation accuracy compared to existing techniques.

## Introduction

The geometric structures present in retinal vascular images, such as vessel diameter, branching angles, and lengths, provide critical information^1^. Ophthalmologists can identify these features to detect and diagnose conditions like hypertension, diabetes, and atherosclerosis^2^. As the world’s population ages and visual impairment increases, the workload of ophthalmologists is also increasing. The increased workload leads to an increased likelihood of human error and increases the health risks of patients. Consequently, there is an urgent requirement for automated retinal vessel segmentation (RVS) in clinical workflows, aimed at minimizing the annotation time and workload of ophthalmologists. This demand has fueled greater interest in developing RVS methods to assist ophthalmologists through computer-aided diagnosis systems.

The primary challenge in RVS arises from the complex and varying anatomical structures of retinal vessels. Thin and low-contrast vessels are particularly difficult to identify, especially in the presence of imaging artifacts or pathological conditions. Existing segmentation methods struggle with accurately detecting and preserving these fine structures while maintaining robustness across different imaging conditions. This paper focuses on addressing these challenges by proposing innovative solutions for automated RVS.

Research into efficient and accurate automatic segmentation methods for fundus images, leading to the development of computer-aided diagnosis technology, holds significant value for the early diagnosis and precise treatment of various eye diseases^3^. Besides, the complex topology of retinal vessels makes their extraction challenging in medical practice. Therefore, developing an efficient and automatic RVS algorithm is of significant importance for clinical pathological diagnosis.

Current RVS methods can be broadly categorized into traditional machine learning and deep learning approaches. Traditional machine learning methods include techniques based on morphological processing^4^, matched filtering^5^, and wavelet transforms^6^. These methods typically do not require prior labeled information but instead analyze the data based on similarities. Marios et al.^7^ constructed a multi-scale line tracking method that utilizes vessel directional properties and morphological reconstruction to achieve RVS. This method reduces the impact of background image noise but fails to fully utilize vascular information, leading to blurred vessel textures. Azzopardi et al.^8^ designed a selective response filter for RVS, which improves the ability to segment crossing vessels but is susceptible to image noise. Jiang ea al.^9^ applied a multi-scale two-dimensional Gabor wavelet transform method to segment retinal images, extracting both thick and thin vessels using different techniques. These methods typically involve preprocessing the retinal image, followed by thresholding to achieve RVS.

Recent advancements have seen the growing integration of deep learning techniques into RVS tasks, resulting in superior segmentation results. The end-to-end learning capabilities of deep learning methods allow for the automatic extraction of both fine-grained and abstract features from images. Compared to traditional segmentation methods, deep learning reduces the need for manual feature extraction and decreases the subjectivity of segmentation, offering significantly better generalization capabilities. For example, fully convolutional networks^10^ achieve pixel-level semantic segmentation by applying convolution, activation functions, and pooling layers in the encoder path, and using convolutional layers and upsampling in the decoder. The encoder-decoder network structure, particularly U-Net^11,12^, has demonstrated remarkable performance in various medical semantic segmentation tasks, including brain tumor segmentation, kidney tumor segmentation, and RVS^13^.

Despite being proposed several years ago, U-Net remains a widely accepted and effective baseline model for medical image segmentation. Its modular and flexible architecture, which combines an encoder-decoder framework with skip connections, facilitates the extraction of detailed spatial information and global contextual features, making it especially suitable for tasks that require precise delineation of fine structures like retinal vessels^14,15^. Additionally, U-Net’s simplicity allows for seamless integration of novel components, such as attention mechanisms, dilated convolutions, and refinement strategies, enabling researchers to adapt and extend its design to meet specific task requirements^16,17^. These qualities make U-Net an ideal foundation for exploring and benchmarking new segmentation techniques, as demonstrated by several recent works^18,19^.

For example, Li et al.^20^ proposed a dynamic-channel graph-based model, which maps retinal image channels into topological space and synthesizes each channel’s features in the topological graph, thereby improving the utilization of retinal vessel information. However, this model overlooks global contextual information, leading to some loss of vessel contour contents in images. Wang et al.^21^ implemented a context-aware network, assigning different weights to channels, enabling the network to fully capture vessel contextual information. However, their model struggles with detecting vessel boundaries, resulting in blurred textures in small vessels. Zhou et al.^17^ employed dense skip connections and introduced convolutions in the skip paths to reduce feature discrepancies between the encoder and decoder. Yue et al.^22^ utilized multi-scale input layers and dense blocks in U-Net, enabling the network to leverage richer spatial contextual information. Moreover, Zhuang^18^ proposed a multi-path U-Net, which constructs multiple paths from input to output using two cascaded U-Nets, achieving higher segmentation accuracy than R2U-Net^23^. Li et al.^19^ proposed a strategy that cascades multiple U-Nets to gradually refine segmentation results, where the output of the previous U-Net is used as the input for the next U-Net, iteratively correcting inconsistent vessels. Yang et al.^24^ designed a spiking neural P-type dual-channel dilated convolutional network that integrates spiking neural convolutional neurons into the classic encoder-decoder structure and uses dilated convolutions in the encoding part to enhance the receptive field. However, these algorithms cannot dynamically adjust the network’s focus area and perform poorly in segmenting vessels in low-contrast regions, such as those affected by lighting variations or lesions. Additionally, they lack precision in segmenting the finer ends of vessels. Moreover, Hong et al.^25^ proposed deep forest framework to address the limitations of deep neural networks in image classification, particularly in scenarios with limited well-curated data. Their method integrates hand-crafted feature extraction and multi-grained scanning, feeding diverse feature representations into different classifiers within a hierarchical deep forest architecture. The framework employs a self-adaptive distance transformation mechanism, wherein prediction vectors at each layer are transformed into distance vectors, which are then fused and concatenated with original features before being input into the subsequent layer. In contrast, our multi-path module introduces a segmentation-specific deep learning architecture tailored for RVS. Instead of relying on a decision-tree-based ensemble model like DTDF-HFF^25^, our method leverages a multi-path U-Net architecture that separately processes texture and structural features through a dual-path framework, allowing for a more refined feature extraction. Furthermore, a CDCM enhances multi-scale feature learning, while a PDA within the upscaling blocks adjusts feature weighting, increasing the contribution of shallow information to improve segmentation in complex backgrounds. Unlike DTDF-HFF, which follows a non-backpropagation-based training paradigm, our approach is trained end-to-end, ensuring joint optimization and enhanced feature representation. These architectural advancements make the proposed model particularly well-suited for medical image segmentation, addressing structural preservation and feature integration more effectively than DTDF-HFF’s classification-oriented methodology.

In this work, U-Net is chosen as the baseline model due to its proven effectiveness in capturing both global and local features, its adaptability for incorporating new modules, and its status as a well-established benchmark in medical image segmentation research^14,26^. The modifications proposed in our model build upon U-Net?s strengths while addressing its limitations, resulting in a robust and effective framework tailored for RVS.

The network is distinguished by three essential features:

- A dual-path U-Net with texture and structure branches, where shared encoder weights enable simultaneous training for segmenting of tiny vessels. The combined segmentation results are refined through a feature refinement module.
- A boosting algorithm that utilizes PDA within the upscaling blocks, dynamically adjusting the network’s focus to emphasize vascular features and reduce overfitting.
- A cascaded dilated convolution module that extracts deep semantic features, guiding the integration of shallow spatial features through long skip connections to restore intricate vascular details.

## Related work

Currently, a significant number of RVS techniques have been developed, falling into two main categories: traditional methods and deep learning-based approaches^27^.

### Traditional segmentation methods

Traditional segmentation models can generally be categorized into region-based segmentation methods, edge detection-based methods, morphology-based methods, active contour model-based methods, and machine learning-based methods^28^. Region-based segmentation methods^29^ primarily based on the similarity of regional features, dividing pixels with strong similarity into the same region. These methods can be further divided into threshold-based methods and region-growing methods. The threshold-based method is simple and commonly used, where the idea is to classify each pixel by comparing it to a selected threshold. This method is widely applied to grayscale image segmentation, but it performs poorly with images that have unclear peaks. Additionally, the choice of threshold significantly affects the segmentation results, making it a crucial aspect of the method. Currently, various methods such as iterative thresholding, histogram thresholding, and Otsu’s method have been proposed. For instance, in ^30^, they first equalize and denoise the image background to approximate a coarse version of the vascular channel using an adaptive local thresholding method, followed by the segmentation of retinal vessels via curvature analysis, also involving morphological opening. Region-growing methods start by creating a region from an initial seed based on some growth criterion, then include neighboring pixels or regions until the condition is met. This method is simple and does not require any prior knowledge, leading to its prevalent use in complex image segmentation tasks. However, the iterative nature of the method incurs a good amount of space and time cost. Furthermore, the selection of seed points is critical to the success of these methods. For example, in ^31^, mage smoothing is accomplished through the use of anisotropic diffusion filters while preserving vascular boundaries, and then RVS is achieved using region-growing and level-set methods.

Edge detection-based methods^32^ primarily rely on the property that significant attribute changes occur at the boundaries between different regions in an image. These methods typically follow a multi-step process. First, they identify edge pixels in the image using specific algorithms. Once identified, these pixels are then connected according to defined rules to form the boundaries of regions. The final step involves detecting edges based on discontinuities in features such as grayscale and texture, thereby achieving image segmentation. The core challenge of these methods, therefore, lies in accurately determining the edge pixels. Various approaches have been designed to tackle this challenge, including the use of image differential operators that apply the first or second derivatives, region-based techniques like the Hough transform, and fitting operator methods that depend on parametric models^33^.

Morphology-based methods mainly use a set of basic morphological operations (e.g., erosion and dilation, opening and closing operations, top-hat transformation) to perform edge detection and feature extraction. In Ref.^34^, a multi-directional and multi-structural element-based morphology is used to separate vessels from their background, followed by morphological operations guided by hysteresis thresholding to eliminate interfering regions in the binary image. In Ref.^35^, an improved morphology combined with Otsu’s method is proposed for unsupervised RVS. First, the top-hat transformation is applied to enhance the contrast between vessels and the background, then a correction method is used to eliminate illumination issues caused by retinal diseases, and finally, Otsu’s thresholding method is used to segment the vessels. Furthermore, active contour model-based methods^36^ achieve vascular segmentation by designing various energy functions that guide the active contour to evolve towards the vessel boundary. While these methods offer high accuracy and stability, they are computationally demanding and therefore not well-suited for large-scale datasets. In Ref.^37^, a new infinite active contour model is proposed, which uses a mixture of regional information from the image.

Machine learning-based vascular segmentation methods focus on defining feature vectors that effectively differentiate vascular structures from other regions in fundus images. Once these feature vectors are established, the next step involves using supervised, unsupervised, or semi-supervised learning algorithms to classify image pixels as either vessel or non-vessel pixels. This classification process ultimately enables accurate vascular segmentation. By combining feature vector definition with the appropriate learning algorithms, these methods can effectively distinguish vascular structures within the images^38^. In Ref.^39^, a supervised retinal vessel extraction method based on multi-feature fusion is proposed. This approach begins by extracting various types of features from the retinal images. These features are then utilized to train a retinal vessel classifier using a random forest algorithm. After the classification step, post-processing is performed to refine the results, incorporating both vascular image grayscale information and connected domain information. This final post-processing step ensures the accurate extraction of the retinal vessels, yielding the final vessel segmentation. In Ref.^40^, a rapid vessel extraction method utilizing extreme learning machines is proposed. However, the method encountered challenges in accurately extracting very small vessels.

### Deep learning-based segmentation methods

With the successful application of deep learning in fields such as natural image classification and natural language processing, deep learning-based image segmentation methods have also emerged. These models mainly use deep learning algorithms to extract deep features from raw images, then combine them with different classifiers to achieve vascular segmentation^41^. A large number of machine learning and deep learning methods are introduced in^42,43^ that briefly reviewed the current chelleges associated with prior methods. In feature encoding-based methods, VGGNet^44^ and ResNet^45^ are two important network architectures. VGGNet is mainly composed of 3$\times$3 convolution kernels and 2$\times$2 max-pooling layers, forming a 16-19 layer deep convolutional neural network. Its advantage is that it solves the parameter explosion problem caused by deepening traditional networks. ResNet?s primary innovation is the implementation of identity mapping, directly transmitting the unprocessed input information to the next layer, and learning only the residuals of the previous network’s output during the process. This addresses the issue of vanishing gradients as the depth of deep learning networks increases. In upsampling-based methods, the Fully Convolutional Network (FCN)^46^ and U-Net^47^ are two representative network models. These methods discard certain features during sampling to preserve more important features, and to some extent, upsampling operations can achieve more precise segmentation boundaries. However, this process is irreversible and may sometimes lead to lower image resolution and loss of details. The FCN approach involves a structure of deconvolution followed by upsampling, with its central concept being the expansion of pixels through sampling, then applying convolution to learn the weights. This approach has the advantage of accepting input images of any size while retaining spatial information, effectively solving the problem of semantic-level segmentation. However, because upsampling is performed on each pixel separately, it fails to account for the relationships between pixels, resulting in spatial inconsistency, some blurred outcomes, and reduced attention to image details. U-Net boost upon FCN with a network model that includes a contracting path for feature extraction and an expanding path for upsampling. The contracting path captures contextual information in the image, while the upsampling part restores the location information of the image. U-Net can train with relatively small data samples to achieve good performance, leading to its prevalent use in medical-related fields. As deep learning continues to evolve, a growing number of deep learning-based methods for vascular segmentation have been introduced^48^. For example, the method^49^ uses an integrated deep learning network of CNN and CRF to delineate retinal vessels. Han et al.^50^ proposed a RVS algorithm that combines DenseNet and U-Net models, improving the accuracy of RVS. Gu et al.^51^ designed a CE-Net o capture richer high-level features for 2D medical image and researchers have been used this idea to implement CNN for quality results in medical images. In short, process medical images has greatly benefited from the successful application of deep learning techniques. due to their ability to capture representative and distinguishable features in images. These deep learning methods have shown an increasing trend in RVS^52^. However, segmenting retinal vessels from fundus images is a challenging task. The main difficulties are: (1) poor contrast and low resolution; (2) image imperfections like noise, blur, and uneven lighting; (3) fluctuations in vessel width and shape; and (4) bifurcation and intersection of vessels^53^. In order to solve the above problems, achieve better automatic segmentation results, and enable these methods to enter a wider range of clinical applications, researchers introduced attention mechanisms in neural networks to elevate the quality of segmentation results. For instance, SE blocks^54^ introduced a lightweight channel attention mechanism that selectively emphasizes useful feature maps, CBAM^55^ combined both channel and spatial attention to improve representation, and Transformer-based attention mechanisms^56^ have recently gained traction for their ability to capture long-range dependencies. While these approaches have shown significant improvements in various tasks, their high computational cost and lack of specialized designs for RVS limit their applicability in this domain. This study presents an enhanced attention-based model specifically designed to address the challenges of RVS.

**Fig. 1:**
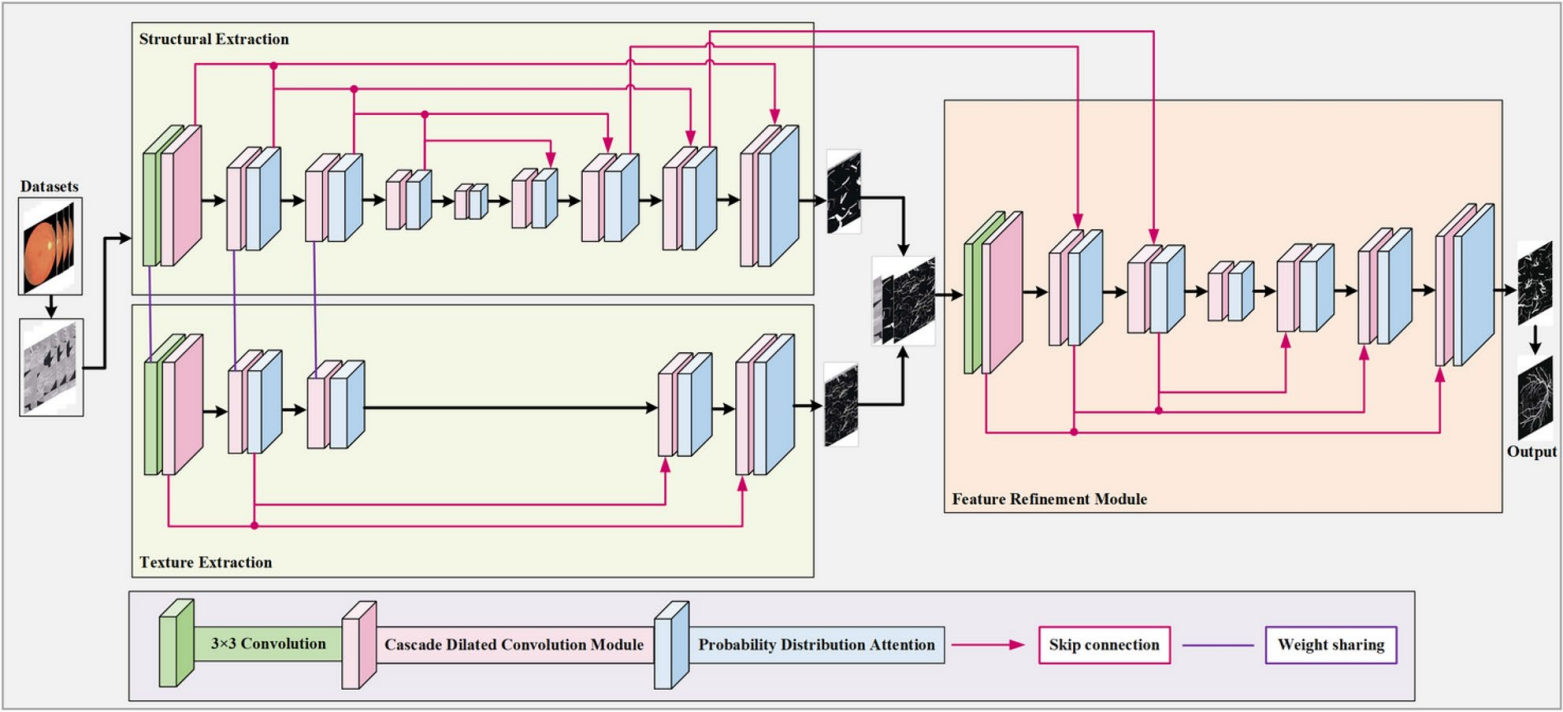
Graphical structure of our model.

## Proposed methodology

### Network structure

Building on the concepts of multi-path U-Net architecture and the iterative refinement strategy from IterNet^19^, we proposed an enhanced multi-path U-Net model. This model is designed by cascading two U-Nets, interconnected through skip connections, creating multiple pathways from input to output. These interconnected paths allow the model to function as a variant of a FCN, facilitating the segmentation of complex and intricate vessel structures. The use of cascading U-Nets enables the model to iteratively refine the segmentation results, improving accuracy with each pass. To further boost the model?s ability to detect and segment fine vessel structures, a shallow U-Net, referred to as the texture branch, is introduced alongside the primary U-Net. This additional branch is specifically designed to capture detailed textural information, complementing the deeper network’s structural segmentation. By incorporating this parallel pathway, the model gains a balanced ability to segment both large vessels and finer, more delicate vessel boundaries. Furthermore, a boosting algorithm that incorporates PDA is integrated within the upscaling blocks of the network. This mechanism helps focus the network’s learning process on relevant regions, mitigating the effects of optical imaging variations and minimizing interference from pathological regions. The attention-guided approach not only sharpens the focus on vascular features but also contributes to the overall robustness and precision of the segmentation process.

As shown in Fig. 1, the network structure is composed of three parts: the structural branch, the texture branch, and the feature refinement module. The structural and texture branches are 5-layer and 3-layer U-shaped networks, respectively, which share encoder weights. Except for the first encoder layer, which consists of sixteen 3$\times$3 convolutions and a residual block, the remaining layers are composed of a casecade dilated convolution moduel followed by an probability distriution atttention module. This setup helps the encoder extract features and aids the decoder in restoring the structural information of fine vessels. Between the encoder layers, downsampling is performed using convolutions with a stride of 2 and kernel size of 2$\times$2 instead of pooling. In the decoder, upsampling is conducted using transposed convolutions with a stride of 2 and kernel size of 3$\times$3. The addition of skip connections between the encoder and decoder, integrated with a dilated convolution module, effectively restores vessel details lost during downsampling. The structural branch segments semantic information that requires extraction by a deep network, such as most of the vessel skeleton. Conversely, the texture branch leverages the shallow network’s capacity for extracting fine-grained details, facilitating the accurate segmentation of delicate vessel boundaries. The preliminary segmentation probability maps from both branches are concatenated with the source image, supplementing features that are not correctly segmented and feeding them into a feature refinement module composed of four U-shaped layers. This module enhances the restoration of missing vessel pixels through feature extraction and integration, thereby refining the initial segmentation results.

Additionally, skip connections are added between the decoder of the structural branch and the encoder of the refinement module, providing additional information at each scale of the feature map and enhancing the feature extraction capability of the refinement module, which also promotes gradient flow. Finally, the final segmentation probability map is output through a Softmax activation function.

### Cascade dilated convolution module

In segmentation tasks, smaller convolution kernels can help detect smaller target areas, while larger convolution kernels can not only detect larger target areas but also eliminate false positive regions. Szegedy et al.^57^ proposed the inception module, which enhances the network’s receptive field by using parallel convolution kernels of different sizes. However, in the CDCM, to avoid the increase in network parameters and the decline in segmentation accuracy caused by dimensionality reduction operations, the 1$\times$1 convolution layer and 3$\times$3 max-pooling layer are removed. Instead, three 3$\times$3 dilated convolutions with different dilation rates are cascaded to capture multi-scale feature information, with dilation rates of 1, 3, and 5, respectively. At last, the resulting feature maps of the three dilated convolutions are fused to extract richer feature information and more extensive abstract features, thereby improving the encoder’s feature representation and reuse capabilities, which facilitates more effective feature representation for larger segmentation targets.

**Fig. 2:**
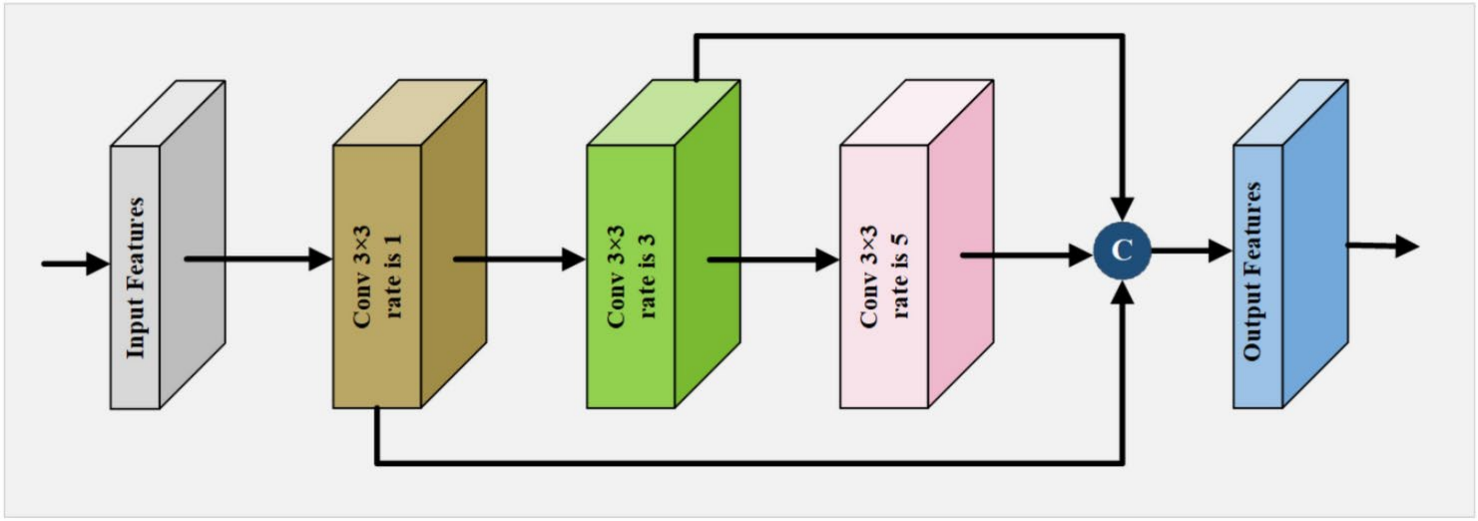
The layout of cascade dilated convolution module.

The combine operation of Conv1$\times$1, BN, ReLU (CBR) is used to reduce dimensions and decrease computation; then, the Conv3$\times$3, BN, ReLU, Dropout (CBRD) operation is applied to learn features and alleviate the overfitting problem. To prevent excessive computational complexity of the model, only the new feature maps generated by the CBRD operation in the previous layer are merged as input for the current layer. Specifically, if the number of output feature maps at each layer is *k*, then the number of input feature maps at the $l^{th}$ layer is $F + (l - 1) \times k$. The output is defined as:

1 $$\begin{aligned} F_l = G([F_0, F_1, \dots , F_{l-1}]) \end{aligned}$$

where $F_l$ represents the output feature map of the l-th layer; G represents the composite function operation of $CBR+CBRD$ and $\bigotimes$ signifies the operation of merging feature maps. Finally, the output feature maps of the four layers are fused with the original feature map and input into the attention module, where they are recalibrated spatially and channel-wise to acquire rich contextual information, encouraging the network to learn more meaningful features. Although this approach increases the number of parameters significantly, literature^58^ substited traditional convolution with dilated convolution to expand the receptive field without inflating the parameter count. However, it does not consider the image boundary effect. Therefore, this paper proposes the CDCM, as shown in Fig. 2, and places it at the bottom across the network.

### Boosting algorithm

Ensemble learning is the method of training weak classifiers to become strong classifiers. Its mechanism involves iteratively refining several base learners and subsequently merging them with weighted summation. The fundamental principle is derived from the literature [26], as outlined below.

Define $\textbf{z} = (z_1, z_2, \dots , z_n)$ as a series of input variables, and *t* as the corresponding target values. Let $\{ (z_i, t_i) \}_{i=1}^n$ be the training samples, and let the loss function be denoted as $\mathscr {L}(t, g(z))$. The decision function *g*(*z*) is optimized over the training samples by minimizing the following loss function:

2 $$\begin{aligned} g^*(z) = \arg \min _{g} \sum _{i=1}^n \mathscr {L}(t_i, g(z_i)) \end{aligned}$$

For simplicity, we choose addition as the combination method between base learners. The model *g*(*z*) is the summation of multiple learning machines:

3 $$\begin{aligned} g(z) = h(z; b_0) + \sum _{k=1}^K \alpha _k h(z; b_k) \end{aligned}$$

Here, $b_0$ and $h(z; b_0)$ represent the initial parameter and the initial base learner, respectively. $b_k$ is the parameter of the *k*-th base learner, $\alpha _k$ is the expansion coefficient, and *K* is the number of base learners. The model *g*(*z*) is optimized step by step. We start by initializing the model with the initial base learner:

4 $$\begin{aligned} b_0 = \arg \min _{b} \sum _{i=1}^n \mathscr {L}(t_i, h(z_i; b)) \end{aligned}$$

5 $$\begin{aligned} g_0(z) = h(z; b_0) \end{aligned}$$

At each iteration *k*, we compute the negative gradient, or residuals, of the loss function evaluated against the model?s present predictions:

6 $$\begin{aligned} r_{ik} = - \left[ \frac{\partial \mathscr {L}(t_i, g_{k-1}(z_i))}{\partial g_{k-1}(z_i)} \right] \end{aligned}$$

We then fit a new base learner $h(z; b_k)$ to the residuals by solving:

7 $$\begin{aligned} b_k = \arg \min _{b} \sum _{i=1}^n \left( r_{ik} - h(z_i; b) \right) ^2 \end{aligned}$$

Next, we compute the expansion coefficient $\alpha _k$:

8 $$\begin{aligned} \alpha _k = \arg \min _{\alpha } \sum _{i=1}^n \mathscr {L}\left( t_i, g_{k-1}(z_i) + \alpha h(z_i; b_k)\right) \end{aligned}$$

We then update the model:

9 $$\begin{aligned} g_k(z) = g_{k-1}(z) + \alpha _k h(z; b_k) \end{aligned}$$

After *K* iterations, the final model is:

10 $$\begin{aligned} g(z) = g_K(z) \end{aligned}$$

### Probability distribution attention

In deep learning, especially for tasks like RVS, the main challenge is effectively integrating shallow and deep features to produce accurate segmentation results. Shallow layers in a neural network capture low-level features like edges and textures, which are essential for outlining the nuanced details of retinal vessels. However, as the network becomes deeper, these shallow features may become diluted or overshadowed by deeper features that capture more abstract, high-level information. This dilution of shallow information can lead to a loss of crucial details, which is particularly problematic in medical imaging tasks where precision is paramount.

PDA addresses this challenge by transforming features into a probability space where the importance of shallow and deep features can be dynamically adjusted^59^. This mechanism ensures that the network does not lose important low-level details while still leveraging the rich contextual information provided by deeper layers. By mapping the features into a probability space, PDA allows for a more refined weighting of these features, ensuring that the final segmentation results are informed by both the fine shallow characteristics and the large-scale contextual information captured by the deeper layers.

Using PDA is especially beneficial in RVS, where accurately delineating blood vessels is crucial. By probabilistically retaining and emphasizing shallow information, PDA refines the model?s segmentation of complex and fine vessel structures, thereby boosting both the accuracy and reliability of the segmentation results. To mitigate the dilution of shallow information, the model proposed in this paper transforms it into a probability space and introduces a PDA. This mechanism adjusts the weight of shallow information after it has been mapped into probability space, continuing this adjustment until the upsampling process is complete. The shallow and deep information in probability space is defined as $\overline{F}_i = P(c,i,\theta )$ and $F_i = P(c,i,\theta )$, respectively.

11 $$\begin{aligned} \overline{F}_i= & P(c,i,\theta ) = \ln \left( \overline{P}_i(X_i = c|I; \theta )\right) \end{aligned}$$

12 $$\begin{aligned} F_i = P(c,i,\theta )= & \ln \left( P_i(X_i = c|I; \theta )\right) \end{aligned}$$

where the size of the image *I* is *N*, *c* represents different segmentation types: the main trunk of the blood vessel, branches, and terminal points, etc. $X_i$ represents the pixel value. Then, using the Softmax function, we can perform probability transformation as:

13 $$\begin{aligned} \text {soft}(F) = \frac{\exp (F_i(c,I,\theta ))}{\sum _{c'}\exp (F_i(c',I,\theta ))} = \frac{P_i(X_i = c|I; \theta )}{\sum _{c'}P_i(X_i = c'|I; \theta )} \end{aligned}$$

**Fig. 3:**
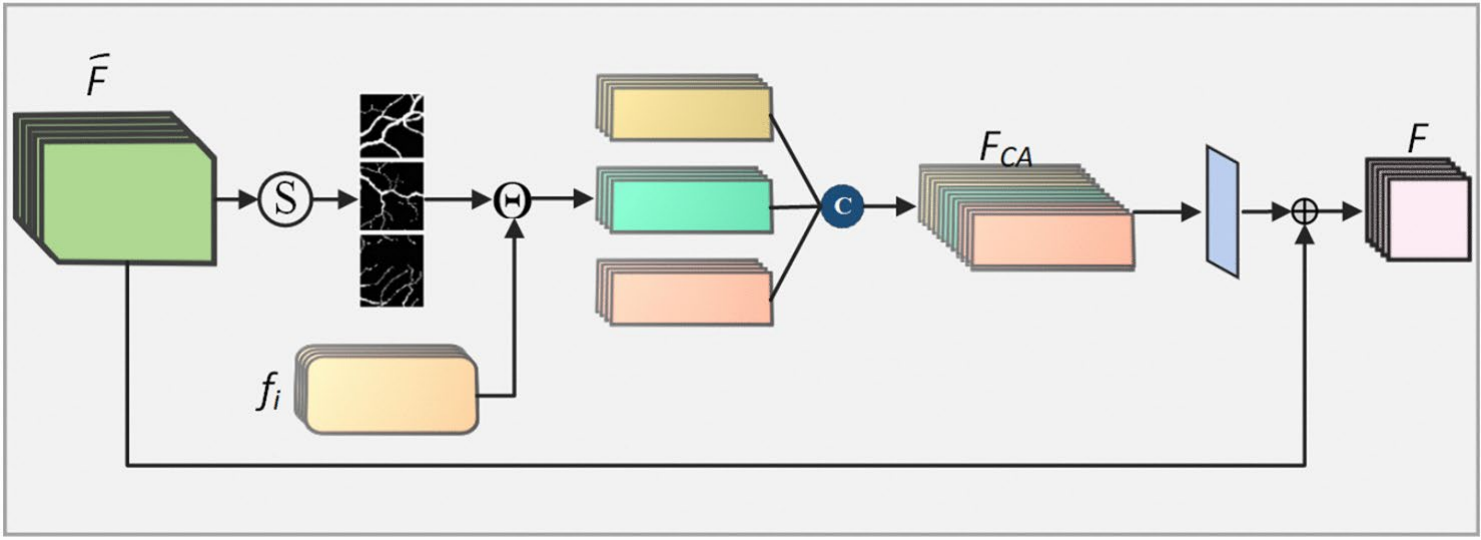
Graphical representation of probability distribution attention.

Shallow information can provide global guidance for the final segmentation. Through equation (13), the segmentation result is introduced into probability space through Softmax, yielding different types of probability maps. Due to the coarseness of the shallow information’s segmentation result, the probability weight corresponding to the main blood vessel trunk is relatively large. For each class probability map, multiply it by each pixel point. After each operation, select the appropriate skip connection, and use the information available before upsampling as $f_i$. At this stage, $f_i$ integrates information from both shallow and deep networks, providing rich shallow semantic details. Then, all attention feature maps of all types are concatenated to obtain $F_{CA}$. In other words, each feature map of $F_{CA}$ is the result of multiplying each single feature map by the corresponding pixel in each type’s probability map. As shown in Fig. 3, *F* is the new vascular feature information obtained after the attention mechanism. The relationship between *F* and $\overline{F}$ is:

14 $$\begin{aligned} \overline{F} = F \oplus \left( \left( \text {soft}(F') \cdot f_i \right) \otimes K \right) \end{aligned}$$

where $\text {soft}(\cdot )$ represents the probability distribution mapping operation, and the multi-class classifier is Softmax.

The proposed PDA is designed to address the shortcomings of existing attention mechanisms by emphasizing both global and local feature representations in RVS. Compared to SE blocks, which focus solely on channel-wise feature recalibration, PDA dynamically integrates shallow and deep features in a probabilistic space, ensuring retention of crucial low-level details. CBAM, while effective for general-purpose tasks, does not fully account for the structural intricacies and low contrast of retinal vessels. Transformer-based attention mechanisms, on the other hand, provide global context but are computationally intensive and prone to overfitting in small medical datasets. PDA strikes a balance by leveraging probabilistic weighting to refine vessel segmentation with minimal computational overhead, making it more suitable for RVS tasks.

Moreover, experimental results as shown in Table 2 and Fig. 10 indicate that PDA outperforms these existing mechanisms in terms of segmentation accuracy, particularly in preserving fine vessel details and handling low-contrast regions. This improvement is attributed to its ability to dynamically adjust the importance of shallow and deep features based on their contribution to the segmentation task.

### Loss function

During the model decoding phase, suppose that the three decoding blocks from left to right are called the lower layer, the middle layer, and the output layer, respectively. Let $\textbf{V}$ be the weights of the network, $\textbf{V}^a$, $\textbf{V}^b$, $\textbf{V}^c$ be the weights of the three decoders, respectively. The cross-entropy loss function for a particular layer can be defined as:

15 $$\begin{aligned} L(Y;\textbf{V}) = \sum _{y_j \in Y} - \text {lb} \, q(t_j | y_j; \textbf{V}, v^d) \end{aligned}$$

where *Y* is the number of training samples, $q(z_j = t(y_j) | y_j; \textbf{V}, v^d)$ is the probability that sample $y_j$ is correctly classified as the corresponding class label $t(y_j)$. $d \in \{a, b, c\}$ is the decoder index, and the resulting loss function is given by:

16 $$\begin{aligned} L(Y; \textbf{V}^a, v^a, \textbf{V}^b, v^b, \textbf{V}^c, v^c) = \sum _{d \in \{a, b, c\}} \beta _d L_d(Y; \textbf{V}, v^d) \end{aligned}$$

where $\beta _a$, $\beta _b$, $\beta _c$ are the weights of the three losses, controlling the strength of different decoders. In this experiment, $\beta _a$, $\beta _b$, $\beta _c$ are set to 0.3, 0.3, and 0.4, respectively. Moreover, the lower and middle layers undergo upsampling to align with the original image size before being concatenated with the output layer. The aggregated loss is calculated using the cross-entropy function.

17 $$\begin{aligned} Loss_{CE} = - \frac{1}{M} \sum _{m=1}^M \left[ h_{m,j} \log q_{m,j} + (1 - h_{m,j}) \log (1 - q_{m,j}) \right] \end{aligned}$$

where *M* represents the number of categories, $q_{m,j}$ is the predicted probability when pixel *j* belongs to the *m*-th category, and $h_{m,j}$ is the true label value corresponding to pixel *j*. The final overall loss of the model is:

18 $$\begin{aligned} Loss = \gamma Loss_{CE} + (1 - \gamma ) \sum _{d \in \{a, b, c\}} \beta _d L_d(Y; \textbf{V}, v^d) \end{aligned}$$

where $\gamma$ is the weight coefficient, and $\gamma$ is set to 0.5 for all experiments in this study.

## Experimental results

### Datasets

**Fig. 4:**
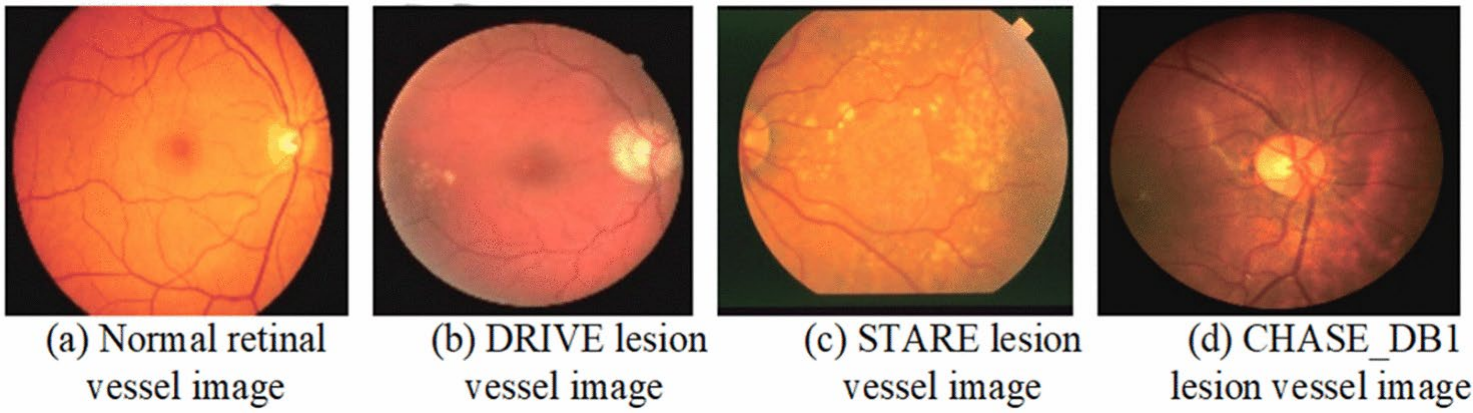
Illustrative examples of retinal vessels and lesions from three public datasets.

In this study, three publicly shared datasets are utilized: DRIVE, STARE, and CHASEDB1. The DRIVE comprises 40 images with a resolution of 584$\times$564 pixels, along with expert-annotated gold standard images. Among these, 7 images exhibit signs of diabetic retinopathy. The official dataset is divided into a training set and a test set, containing 20 images each. The STARE dataset consists of 20 fundus images with a resolution of 605$\times$700 pixels and corresponding expert-annotated gold standard images. Image samples are shown in Fig. 4. Since the official dataset does not provide a predefined split, this study follows the division method used in the DRIVE dataset, where the first 10 images are assigned to the training set and the remaining 10 images to the test set. The CHASEDB1 dataset includes 28 fundus images with a resolution of 999$\times$960 pixels, along with expert-annotated gold standard images. The official dataset provides a division where the first 20 images form the training set and the remaining 8 images constitute the test set. This consistent approach to dataset division across all three datasets ensures a standardized evaluation framework, enabling a fair comparison of model performance across different datasets. The characteristics of common public datasets are compared in Table 1.

**Table 1:** Details of three datasets and augmentation Dataset details extracted from publicly available medical image repositories.

No. of Datasets	No. of Images	Types of Images	Viewing Orientation	Patch-Based Training Samples	Patch-Based Validation Samples	Testing Images
DRIVE	40	JPEG	45	180,000	20,000	20 images
STARE	20	PPM	35	90,000	10,000	10 images
CHASEDB1	28	TIFF	30	140,000	10,000	14 images

### Data preprocessing

**Fig. 5:**
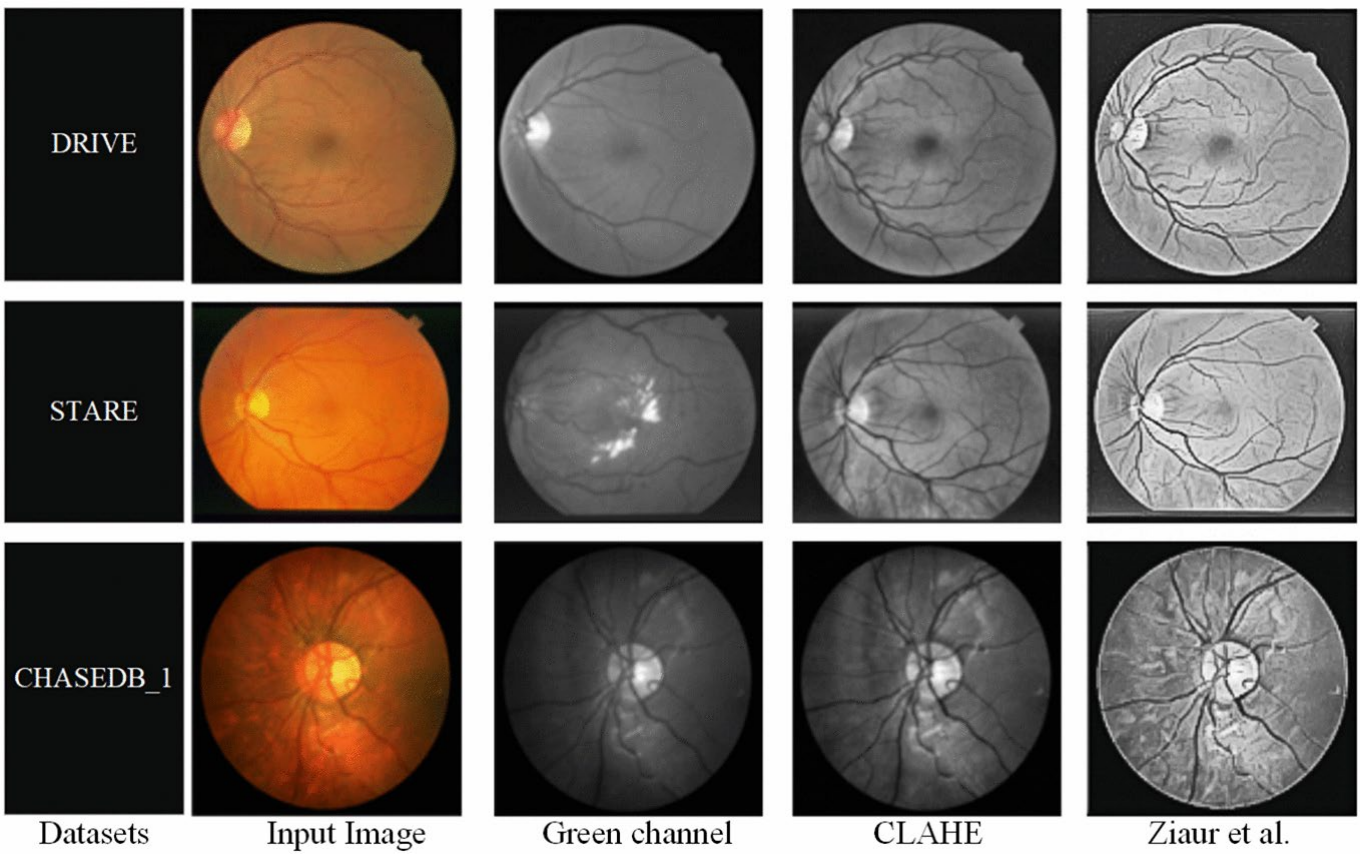
Visual depiction of the preprocessing step.

To mitigate the difficulties associated with low contrast between vessel foregrounds and non-vessel backgrounds in the dataset images, preprocessing step is crucial for accurately capturing retinal vessel features and improving segmentation performance. As illustrated in Fig. 5, the preprocessing pipeline adopted in this study consists of several key steps: the process starts with the conversion of the images to grayscale, reducing memory usage and computational complexity while enhancing the visibility of target regions. Subsequently, the grayscale images are normalized to ensure consistent pixel intensity distribution, mitigating brightness variations that could negatively impact segmentation. After normalization, CLAHE^60^ is applied to the grayscale images to counteract uneven illumination, improving contrast in localized regions and facilitating vessel-background differentiation.

The choice of CLAHE is guided by its proven ability to enhance local contrast in medical images, as it operates on small regions of an image, ensuring that even subtle features like thin retinal vessels become more distinguishable. Other contrast enhancement methods, such as histogram equalization (HE) and adaptive histogram equalization (AHE), are considered but were found to either over-enhance global features or amplify noise in darker regions. CLAHE, in contrast, provides a balanced enhancement, preserving both global structure and local details critical for segmentation.

Lastly, before inputting the images into the network model, preprocessing is conducted using a model outlined by Ziaur et al.^61^, which specifically enhances the visibility and involving the optic disc and vascular elements, particularly in darker regions, resulting in optimized segmentation accuracy. Extensive experiments confirmed that the preprocessing pipeline, including CLAHE, significantly improved segmentation metrics (PSNR, SSIM) by approximately 10-15% compared to using raw images directly. However, care is taken to assess potential artifacts introduced by CLAHE, and it was found that any such artifacts are negligible and did not adversely affect segmentation performance.

### Data augmentation and parameter settings

**Fig. 6:**
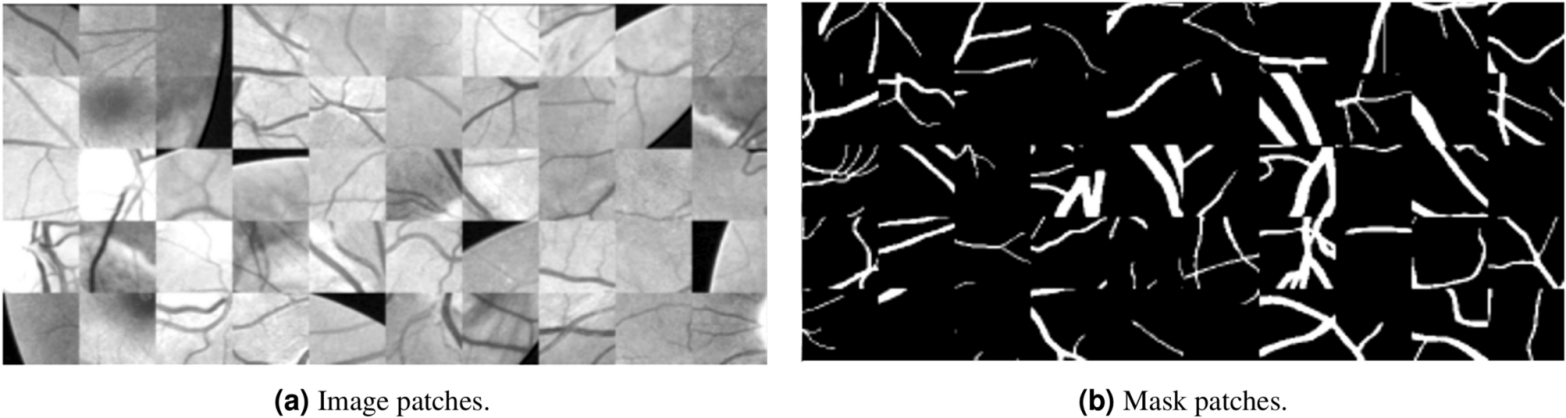
Image patches and corresponding mask patches.

To accurately fit the model parameters, training deep learning networks requires a substantial amount of data. However, the DRIVE, CHASE_DB1, and STARE datasets are relatively small, and a limited training set may not sufficiently capture the statistical characteristics of the entire dataset. In datasets section we mentioned about the number of images in each dataset. The insufficiency of data can cause overfitting, meaning the model may perform exceptionally well on training data yet falter when confronted with new data. To mitigate this issue, image augmentation is performed using a sliding window approach. The preprocessed fundus images and their corresponding ground truth images are cropped into 10,000 feature image patches of size 64$\times$64 pixels using the sliding window method. This technique not only increases the number of training samples but also ensures that the model is exposed to diverse features, improving its generalization ability. The cross-entropy function was employed as the loss function, with an initial learning rate set to 0.0005. The batch size was configured to 6, and the number of epochs is set to 20. An early stopping mechanism was implemented during training, where the training process would be halted if no best weights are saved for 10 consecutive epochs. This approach prevents overfitting and ensures optimal model performance. Regarding computational efficiency, the training time per epoch for the STARE dataset was 1 minute 16 seconds, and the testing time was 52 seconds. For the DRIVE dataset, the training time per epoch was 1 minute 17 seconds, with a testing time of 2 minutes 48 seconds. Similarly, for the CHASE_DB1 dataset, the training time per epoch was 1 minute 17 seconds, and the testing time was 3 minutes 53 seconds. Figure 6aillustrates the preprocessed local feature image patches and their corresponding ground truth patches from the training set.

Furthermore, data augmentation techniques is employed to synthetically expand the dataset and enhance the model’s generalizability. The employed techniques encompassed random rotations, flips, and adjustments in image scaling. Moreover, a sliding window approach is implemented, allowing for the extraction of 48$\times$48 patches from each image. This strategy ensures the comprehensive capture of both global and local vessel structures during the training process.

### Transfer learning

We utilized transfer learning to accelerate convergence and enhance the performance of our model. In this approach, the encoder initialization is performed with pre-trained weights derived from the ImageNet dataset^62^. These pre-trained weights provide a solid foundation for the model by incorporating knowledge gained from millions of diverse natural images, enabling the extraction of basic characteristics, including textures, edges, and structural shapes. This initial knowledge helps the model to recognize basic image patterns more efficiently, setting the stage for specialized training. After this initialization, the encoder undergoes a fine-tuning process using RVS datasets. During this phase, the encoder’s weights are gradually adjusted to better align with the specific characteristics of retinal images, while the decoder is trained from scratch to learn features unique to the task. The fine-tuning process allows the model to adapt the generalized features learned from natural images to the more specific domain of RVS. This adaptation not only adjust the network’s ability to identify vascular structures but also results in a significantly faster convergence and enhanced segmentation accuracy. By leveraging transfer learning, the model achieves a more effective balance between utilizing existing knowledge and adapting to new, domain-specific features.

## Evaluation metrics

Retinal fundus images are classified into two categories: vessel foreground and non-vessel background. The vessel foreground refers to the target vessels that need to be detected and segmented, called the positive class, while the non-vessel background refers to the remaining areas, called the negative class. To clearly demonstrate the superior performance of the algorithm presented in this paper, the segmentation results of the algorithm are compared with the manual segmentation results of experts.

For vessel foreground pixels, the proportion of the algorithm’s segmentation results that match the expert manual segmentation is referred to as the True Positive (TP), while the opposite is referred to as the False Positive (FP). For non-vessel background pixels, the proportion of the algorithm’s segmentation results that match the expert manual segmentation is referred to as the True Negative (TN), while the opposite is referred to as the False Negative (FN). In this study, the specific formulas for Accuracy (Acc), Sensitivity (Sen), F1-score (F1), Specificity (Spe), and the Area Under the Receiver Operating Characteristic Curve (AUC) are as follows:

19 $$\begin{aligned} Acc= & \frac{TP + TN}{TP + TN + FP + FN} \end{aligned}$$

20 $$\begin{aligned} Sen= & \frac{TP}{TP + FN} \end{aligned}$$

21 $$\begin{aligned} F1= & \frac{2TP}{2TP + FP + FN} \end{aligned}$$

22 $$\begin{aligned} Spe= & \frac{TN}{TN + FP} \end{aligned}$$

### Qualitative and quantitative performance analysis

**Fig. 7:**
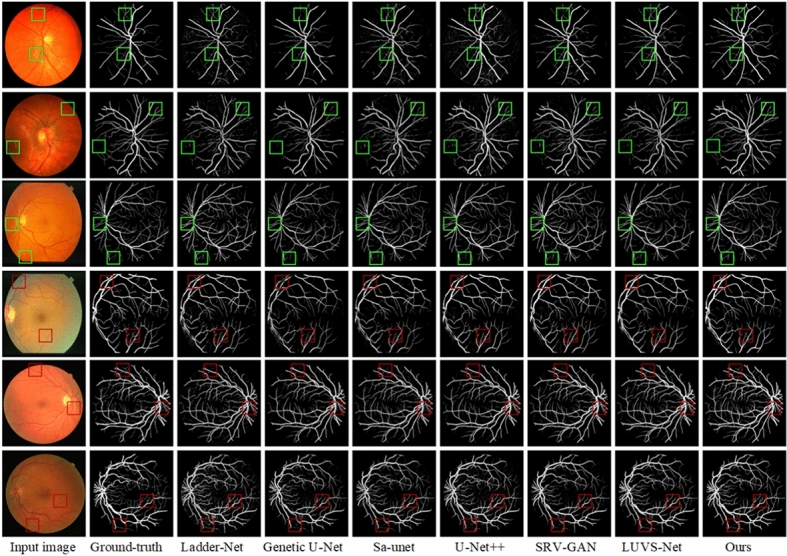
Comparison of blood vessel segmentation of proposed and prior algorithms using $CHASEDB1$, START, and DRIVE datasets.

To provide evidence of the superiority of the proposed model, we conducted several experiments on three datasets and compared our approache with other state-of-the-art algorithms. First experiment is summarized in Fig. 7 that presents the vessel segmentation results of various algorithms. Rows 1 and 2 correspond to images from the CHASE_DB1 dataset, rows 3 and 4 to images from the STARE dataset, and rows 5 and 6 to images from the DRIVE dataset. Figure  7(c) through  (i) represent the results of Ladder-Net^18^, Genetic U-Net^63^, Sa-unet^64^, U-Net++^17^, SRV-GAN^65^, LUVS-Net^66^ and our proposed algorithm, respectively.

In the first row of healthy images, it is evident that other models experience issues such as broken vessels and blurred vessel trajectories when segmenting small vessels. In contrast, our algorithm demonstrates stronger robustness in segmenting small vessels, with more precise and clearer vessel boundaries. In the second row of pathological images, other models show interruptions in small vessel branches, along with missegmentation of pathological information and noise as vessels. However, our algorithm exhibits fewer interruptions in small vessel branches and effectively suppresses the missegmentation of pathological information and noise.

**Fig. 8:**
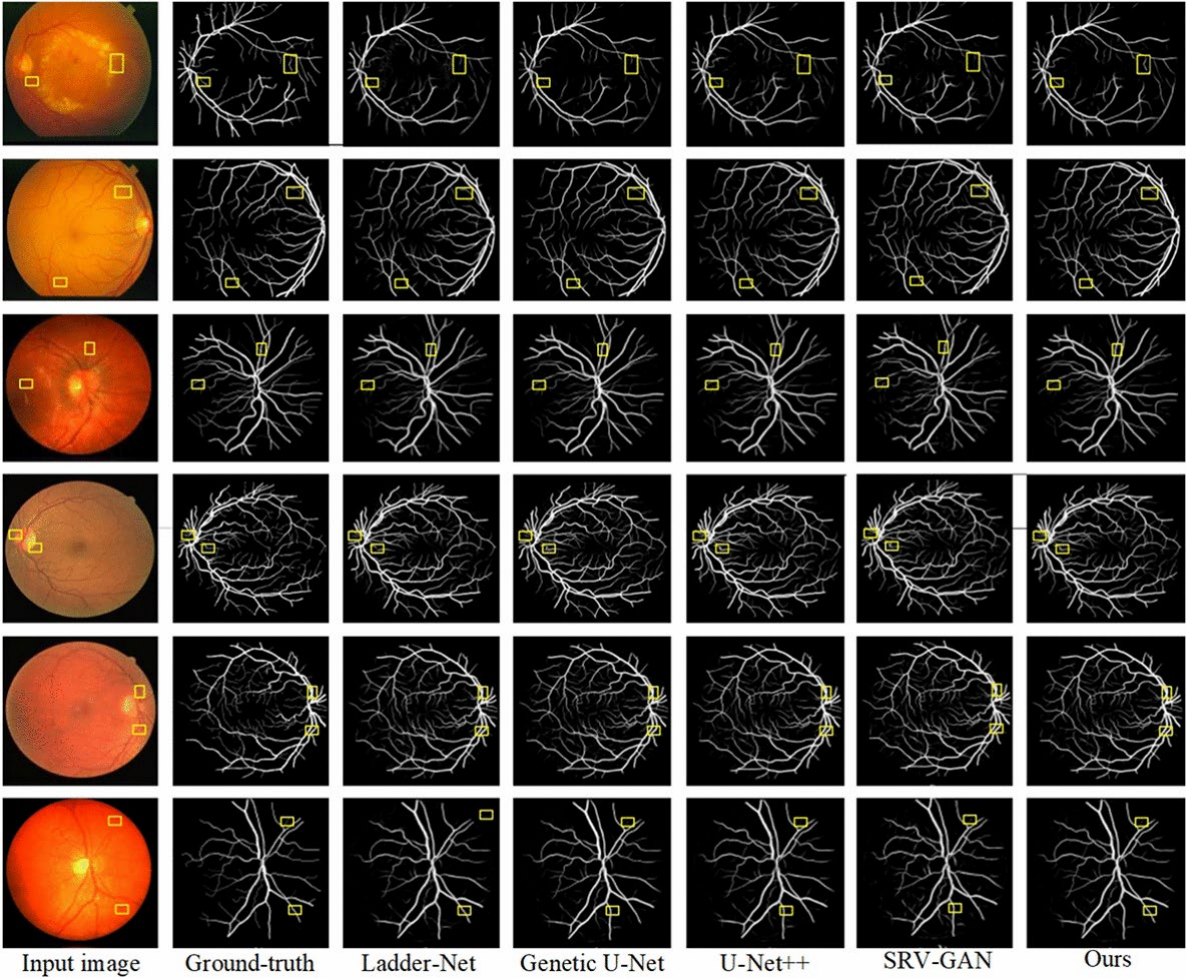
Visual comparison of RVS of our model and exisiting methods using $CHASEDB1$ dataset.

In the third row of healthy images, Ladder-Net^18^, Genetic U-Net^63^, and Sa-unet^64^ fail to extract contours and thin delicate vessels, while other models show segmentation breaks in major vessels. Our algorithm successfully segments both major and thin vessels, providing clearer results even in vessels with blurred boundaries. The fourth row of pathological images reveals that Ladder-Net^18^, Genetic U-Net^63^, Sa-unet^64^, SRV-GAN^65^ and U-Net++^17^ generate significant noise in their segmentation results and misclassify pathological information as vessels, leading to higher false positive rates. Both our algorithm and LUVS-Net^66^ effectively suppress the interference of artifacts and the missegmentation of pathological information.

In the fifth row of healthy images, our algorithm achieves more complete segmentation of both major and small vessels, ensuring smooth connections between the ends of small vessels and the main vessels. Other models exhibit missing ends of small vessels and breaks in the main vessels near the optic disc. In the sixth row of pathological images, the influence of image artifacts and pathological regions causes Ladder-Net^18^, Genetic U-Net^63^, and Sa-unet^64^ to show breaks at vessel intersections. Although our algorithm’s segmentation results contain minor missegmentations, it still outperforms other algorithms in clearly segmenting vessel intersections, particularly between major and small vessels. Based on the above analysis, our algorithm demonstrates a superior ability to accurately differentiate vascular elements from the background in retinal images, while also extracting detailed vessel information more completely and accurately. Furthermore, our algorithm performs better in both healthy and pathological image segmentation tasks, highlighting its robustness.

Furthermore, Fig. 8 presents the results of our second experiment on vessel segmentation, comparing performance across the CHASE_DB1, DRIVE, and STARE datasets. The first two rows show images from the CHASE_DB1 dataset, the third and fourth rows from DRIVE, and the fifth and sixth rows from STARE.

In the first row, which features healthy retinal images, our algorithm demonstrates strong performance by effectively suppressing pathological information. In contrast, U-Net++^17^ exhibits vessel breakage when segmenting major vessels, while Ladder-Net^18^ and Genetic U-Net^63^ show incomplete segmentation of small vessels. SRV-GAN^65^ is significantly affected by pathological artifacts, resulting in a high false positive rate due to misclassification of pathological information as vessels. The second row, displaying pathological retinal images, highlights further weaknesses in U-Net++^17^ and Ladder-Net^18^, which both show fragmentation and gaps in small vessel segmentation. Genetic U-Net^63^ and SRV-GAN^65^ also fail to capture much of the small vessel information, leading to reduced sensitivity.

**Fig. 9:**
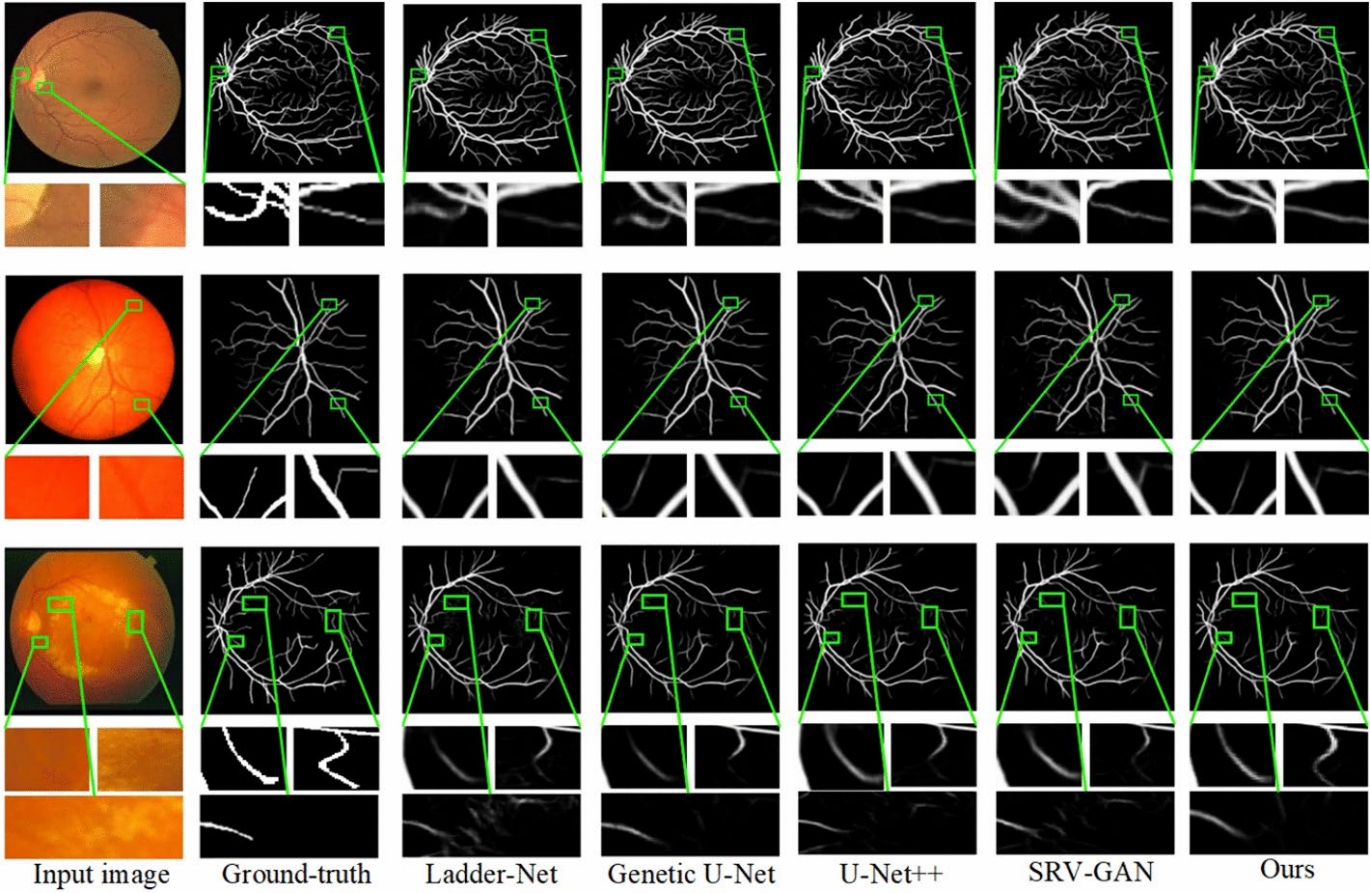
Visual results of our approach and previous models via STARE and CHASE_DB1.

**Fig. 10:**
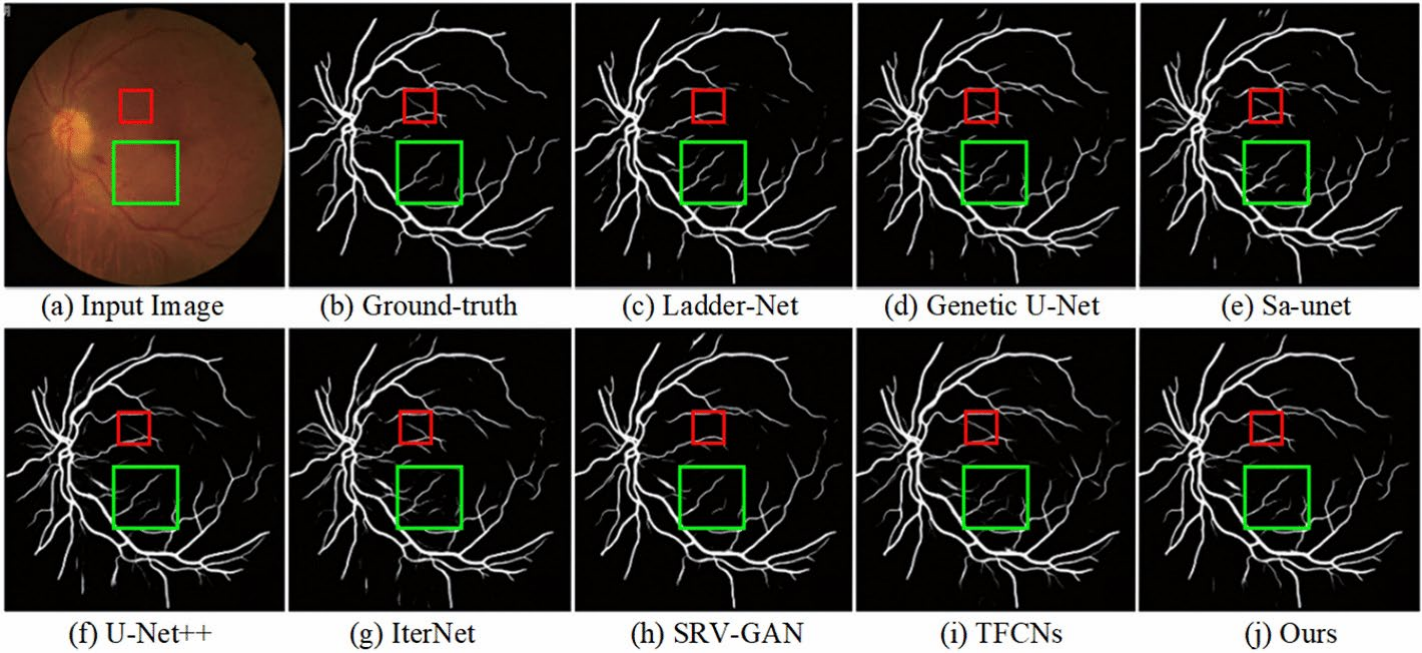
Segmentation of thin and low-contrast vessels in retinal images.

In the third and fourth rows, which focus on the DRIVE dataset, all algorithms performed well in segmenting major vessels. However, small vessel segmentation proved challenging, with only our algorithm avoiding significant missegmentation or missing small vessels altogether. The fifth row from the STARE dataset demonstrates that our algorithm accurately segmented a greater number of small vessels with fewer breakages and correctly distinguished vessels from the background. Finally, the sixth row, which presents pathological images from STARE, shows that U-Net++^17^, Genetic U-Net^63^, and SRV-GAN^65^ misidentified pathological information as vessels, creating clutter around the vessels due to artifacts. In contrast, our algorithm effectively removed pathological spots and minimized vessel loss. Overall, these results confirm that our proposed algorithm excels in RVS by accurately capturing vessel morphology with fewer false positives. This leads to clearer, less noisy segmentation images, reinforcing the method’s effectiveness and reliability.

Figure 9 illustrates the segmentation results across various datasets. In the first row, showing images from the DRIVE dataset, Ladder-Net^18^ struggles with vessel continuity, as its segmentation of major vessels shows noticeable breakage. Genetic U-Net^63^?s performance is hindered by the influence of pathological artifacts, resulting in poor differentiation of non-vessel background areas. U-Net++^17^ encounters issues with incomplete segmentation of small vessels and breaks in vessel continuity. Both SRV-GAN^65^ and the proposed method deliver better overall results; however, our method achieved smoother connections between major and small vessels compared to SRV-GAN^65^.

**Table 2:** Segmentation evaluation of our model against prior algorithms on low-contrast vessels in retinal images.

Methods	Year	$A_{cc}$	*SE*	*SP*	$F_{1}$ score	AUC
DUNet^67^	2019	0.9846	0.8858	0.9837	0.8889	0.9922
DCU-net^68^	2022	0.9845	0.8905	0.9923	0.8894	0.9934
SRV-GAN^65^	2022	0.9834	0.8991	0.9899	0.8923	0.9928
LUVS-Net^66^	2023	0.9844	0.8977	0.9911	0.8915	0.9932
ARDC-UNet^69^	2024	0.9846	0.8974	0.9929	0.8906	0.9936
ResMU-Net^70^	2024	0.9857	0.8943	0.9942	0.8959	0.9926
**Ours**	–	**0.9869**	**0.8984**	**0.9925**	**0.9023**	**0.9932**

In the second row, focusing on small vessels in the CHASE_DB1 dataset, the magnified comparisons revealed that Ladder-Net^18^, Genetic U-Net^63^, U-Net++^17^, and SRV-GAN^65^ all exhibit a loss of vessel texture information. Additionally, small vessels are either segmented with breaks or not segmented at all, depending on the algorithm.

The images in third row is taken from the STARE dataset. It shows that both Ladder-Net^18^ and Genetic U-Net^63^ suffer from vessel breakage. Furthermore, except for the proposed method, all other algorithms demonstrate incomplete segmentation of small vessels. Upon closer inspection of the vessel boundaries, it is evident that Ladder-Net^18^, U-Net++^17^, and SRV-GAN^65^ are significantly affected by pathological information, leading to cluttered regions around the vessels. In contrast, our method and U-Net++^17^ effectively remove pathological spots and elevate the quality and segmentation accuracy.

The experiment focuses on segmenting fine and low-contrast vessels in retinal images, a critical challenge in medical imaging. Figure 10 presents a comparative analysis of the visual results produced by the proposed method alongside several prior models. Notably, Ladder-Net^18^ and Genetic U-Net^63^ exhibit significant vessel breakage, failing to capture the continuity of thin vessels. Additionally, other algorithms show incomplete segmentation, particularly of smaller vessels, highlighting limitations in detecting fine structures. A closer examination of the vessel boundaries reveals that methods such as Ladder-Net^18^, U-Net++^17^, IterNet^19^, and SRV-GAN^65^ are adversely affected by pathological features, resulting in cluttered regions surrounding the vessels. This reduces the clarity and accuracy of segmentation in areas affected by disease. In contrast, the proposed method and TFCNs^71^ demonstrate superior performance, effectively removing pathological artifacts and delivering cleaner segmentation results. These findings underscore the robustness of the proposed approach in improving segmentation quality, especially in challenging pathological contexts.

In short, the proposed method demonstrates notable improvements over other algorithms. It captures vessel semantic information more effectively, allowing for the segmentation of a greater number of small vessels, even in images with severe pathology and low contrast. The segmentation results closely align with the ground truth, highlighting the robustness of our approach in RVS.

To further demonstrate the performance of the proposed method in vessel segmentation, it is compared with state-of-the-art algorithms from recent years across three datasets, as shown in Tables2,  3,  4, and  5. The bold values indicate the best performance for each respective metric. The quantitative evaluation of the our method demonstrates superior performance when compared to various established algorithms. These results provide a clear indication of the advancements achieved by our method in RVS.

**Table 3:** Quantitative analysis of our model against prior algorithms using DRIVE.

Methods	Year	$A_{cc}$	*SE*	*SP*	$F_{1}$ score	AUC
DUNet^67^	2019	0.9576	0.8063	0.9810	0.8337	0.9812
CE-Net^51^	2019	0.9533	0.9853	0.8368	0.9087	0.9566
HAN^72^	2020	0.9591	0.8091	0.9823	0.8393	0.9833
AG-UNET^73^	2020	0.9868	0.8041	0.9808	0.8316	0.9857
RNA-Net^74^	2020	0.9702	0.8127	0.9862	0.8306	0.9860
Spider U-Net^75^	2021	0.9707	0.8119	0.9868	0.8326	0.9876
MAN-RVS^76^	2021	0.9591	0.8146	0.9815	0.8403	0.9837
LACNN^77^	2021	0.9583	0.7835	0.9848	0.8305	0.9826
DCU-net^68^	2022	0.9578	0.8215	0.9790	0.8372	0.9820
Bridge-Net^78^	2022	0.9575	0.7953	0.9828	0.8303	0.9844
LUVS-Net^66^	2023	0.9588	0.9837	0.8384	0.9077	0.9773
ARDC-UNet^69^	2024	0.9714	0.8019	0.9885	0.8341	0.9882
ResMU-Net^70^	2024	0.9709	0.8060	0.9876	0.8325	0.9879
**Ours**	–	**0.9880**	**0.9855**	**0.9900**	**0.9100**	**0.9920**

**Table 4:** Performance evaluation of our method against prior algorithms using CHASE_DB1.

Methods	Year	$A_{cc}$	*SE*	*SP*	$F_{1}$ score	AUC
DUNet^67^	2019	0.9674	0.8175	0.9851	0.8378	0.9882
CE-Net^51^	2019	0.9531	0.9882	0.8095	0.8934	0.9635
HAN^72^	2020	0.9680	0.8339	0.9823	0.8291	0.9881
AG-UNET^73^	2020	0.9677	0.8232	0.9850	0.8393	0.9903
LACNN^77^	2020	0.9665	0.8070	0.9833	0.8173	0.9861
RNA-Net^74^	2020	0.9666	0.8078	0.9828	0.8131	0.9849
Spider U-Net^75^	2021	0.9691	0.9888	0.8093	0.8936	0.9623
MAN-RVS^76^	2021	0.9620	0.8255	0.9762	0.7983	0.9814
DCU-net^68^	2022	0.9618	0.8276	0.9714	0.7992	0.9875
Bridge-Net^78^	2022	0.9683	0.8502	0.9811	0.8348	0.9884
LUVS-Net^66^	2023	0.9763	0.8251	0.9864	0.8100	0.9905
ARDC-UNet^69^	2024	0.9682	0.9921	0.8349	0.9104	0.9689
ResMU-Net^70^	2024	0.9754	0.8128	0.9870	0.8085	0.9886
**Ours**	–	**0.9780**	**0.9930**	**0.9885**	**0.9120**	**0.9920**

**Table 5:** Performance evaluation of our approach against prior algorithms using STARE.

Methods	Year	$A_{cc}$	*SE*	*SP*	$F_{1}$ score	AUC
DUNet^67^	2019	0.9678	0.8102	0.9874	0.8389	0.9910
CE-Net^51^	2019	0.9333	0.9252	0.8031	0.8636	0.9446
HAN^72^	2020	0.9745	0.8051	0.9893	0.8316	0.9866
AG-UNET^73^	2020	0.9683	0.8286	0.9854	0.8479	0.9891
LACNN^77^	2020	0.9711	0.7815	0.9896	0.8246	0.9891
MAN-RVS^76^	2021	0.9651	0.7695	0.9888	0.8243	0.9842
RNA-Net^74^	2020	0.9651	0.7918	**0.9913**	0.8521	0.9923
Spider U-Net^75^	2021	0.9650	0.7698	0.9888	0.8242	0.9834
DCU-net^68^	2022	0.9675	0.8014	0.9880	0.8376	0.9874
SRV-GAN^65^	2022	0.9765	0.8068	0.9915	0.8436	0.9919
LUVS-Net^66^	2023	0.9431	0.9655	0.8304	0.8966	0.9395
ARDC-UNet^69^	2024	0.9761	0.8084	0.9906	0.8399	0.9911
ResMU-Net^70^	2024	0.9623	**0.9718**	0.9299	0.8989	0.9573
**Ours**	2021	**0.9780**	0.9710	0.9900	**0.8500**	**0.9930**

Firstly, our method outperformed all prior algorithms and achieved the highest accuracy. Compared to its closest competitors, such as ARDC-UNet and ResMU-Net, our method shows significant improvements in both sensitivity and F1-score, demonstrating its ability to accurately identify vessels while minimizing false positives. This highlights the robustness of our technique in detecting retinal vessels under varying conditions and complexities in the DRIVE dataset.

Secondly, the evaluation on CHASE_DB1 further solidifies the strength of our method and achieved the best overall performance with an accuracy of 0.9780, sensitivity of 0.9930, specificity of 0.9885, F1-score of 0.9120, and an AUC of 0.9920. This performance is notably superior to ARDC-UNet and LUVS-Net, where our method exceled in sensitivity and F1-score, ensuring a more precise balance between true positives and false negatives. These results suggested that proposed model is highly reliable in segmenting fine details within retinal images, which is crucial for medical diagnoses.

Thirdly, our method once again achieved the highest accuracy (0.9780), F1-score (0.8500), and AUC (0.9930), surpassing even strong contenders like SRV-GAN and RNA-Net. Although RNA-Net has a marginally higher specificity (0.9913), ours’ superior performance in F1-score and AUC underscores its balanced and consistent performance across various metrics, indicating that it is not only effective in segmenting retinal vessels but also efficient in minimizing errors. In short, our model consistently outperformed the prior approaches, especially in critical metrics such as accuracy, F1-score, and AUC. This confirms that our proposed method sets a new benchmark for RVS, offering significant improvements in precision and reliability over prior methods. These results highlight that our method has the potential for application in clinical environments, where accurate and efficient RVS is essential.

**Fig. 11:**
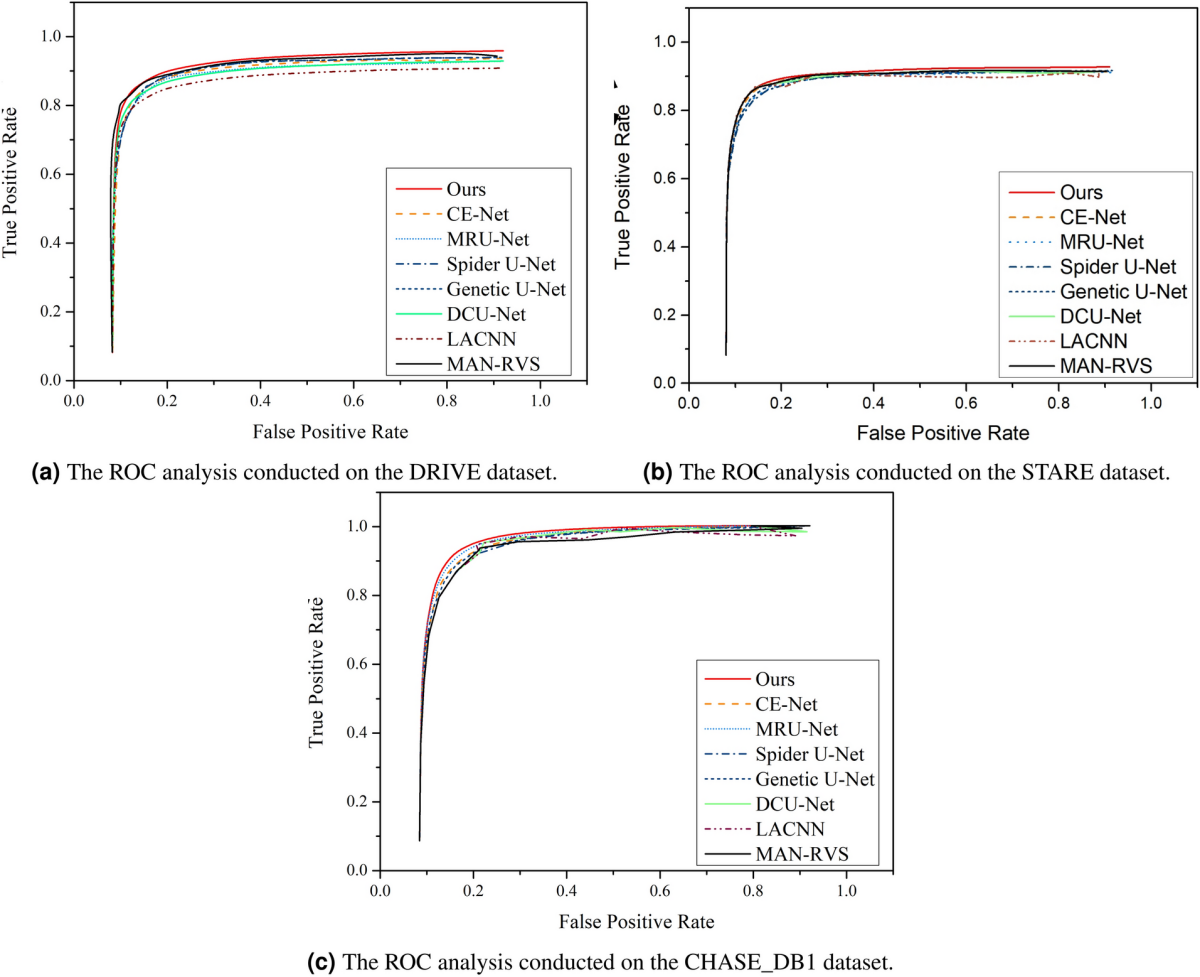
Analysis of ROC curve between our model and prior approaches using DRIVE, STARE, and CHASE_DB1.

Furthermore, Fig. 11 illustrate the comparison of ROC curves for different algorithms on the DRIVE, STARE and CHASE_DB1 datasets. These curves provide valuable insights into the performance of the algorithms in question. The AUC metric that is associated with the ROC curve, particularly important in this context. The AUC metric falls within the range of 0 to 1, with values near 1 indicating superior predictive ability of the model. In other words, the higher the AUC, the more accurate the model is in distinguishing between different classes. Conversely, a lower AUC value suggests a higher rate of misclassification, implying that the model’s performance is less reliable. Thus, by examining these curves and their respective AUC values, we can better understand and compare the effectiveness of the algorithms under consideration.

**Fig. 12:**
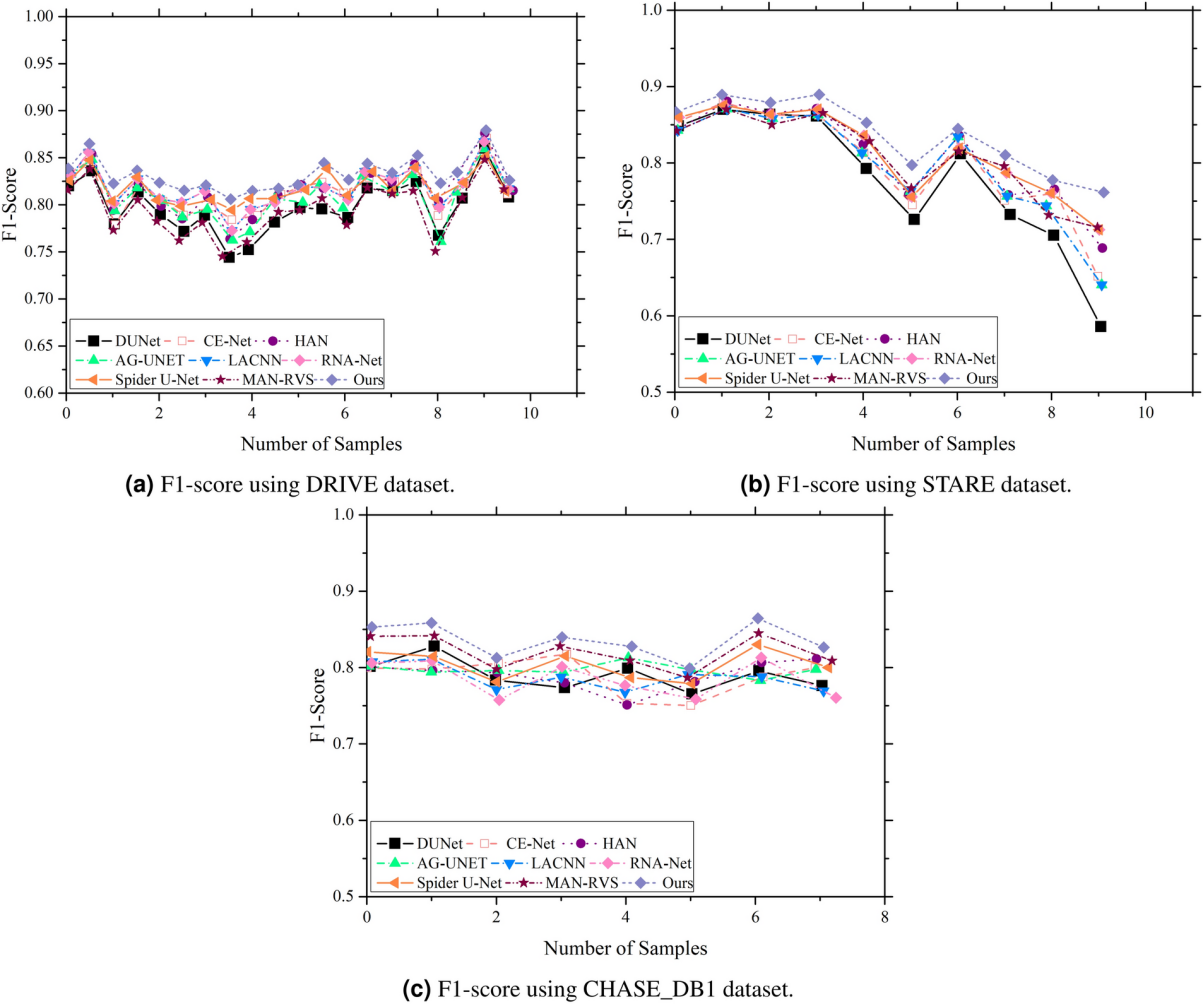
Performance comparison of F1-score between our model and existing approaches using DRIVE, STARE and $CHASE\_DB1$.

To further analyze the performance of different algorithms on individual image segmentation, Fig. 12a and c revealed that our model achieved higher F1-scores than other advanced algorithms. The F1-score curve exhibits smaller fluctuations and is relatively smoother, demonstrating that the proposed method can consistently extract feature information across different datasets. Moreover, it maintains stable segmentation performance for both healthy and diseased retinal images, highlighting the strong generalization ability of the model.

In short, our model has significant clinical relevance and is highly practical for adoption, especially in managing a variety of pathologies and diverse imaging conditions. Our proposed is designed to enhance RVS across various clinical scenarios. From a clinical perspective, accurate segmentation of retinal vessels is critical for the early detection and diagnosis of vision-threatening diseases such as DR, glaucoma, and hypertensive retinopathy. Our method’s ability to capture fine-grained and coarse vessel structures through its dual-path architecture allows for robust segmentation even in cases of pathological variations, such as microaneurysms, hemorrhages, or vessel occlusions, which are commonly seen in DR. Additionally, the PDA mechanism ensures better segmentation in low-contrast regions, which is particularly beneficial when dealing with fundus images of patients with media opacities or poorly dilated pupils.

Regarding practical adoption, our approach demonstrates strong generalizability across different imaging conditions. The CDCM enables multi-scale feature learning, making it well-suited for handling variability in fundus imaging modalities. Furthermore, our model is designed to mitigate overfitting, ensuring that it performs reliably across datasets acquired from different imaging devices, clinical centers, and patient demographics. This adaptability is crucial for real-world deployment in automated screening systems or clinical decision support tools, where models must handle heterogeneous data.

To further highlight these aspects, we have enhanced this study by elaborating on the clinical relevance of our segmentation results, providing specific examples of how our method performs in challenging pathological cases, as depicated in Figs. 9 and 10. The feasibility of integrating our approach into AI-assisted diagnostic workflows is promising, highlighting its potential to improve early disease detection, reduce manual annotation efforts, and enhance diagnostic consistency in ophthalmology clinics. This integration could streamline clinical processes, increase diagnostic accuracy, and ultimately lead to better patient outcomes.

### Ablation study

To validate the contributions of the individual components in the proposed method, we conduct ablation experiments to systematically analyze their impact on segmentation performance. The experiments are performed on randomly selected images from the DRIVE, STARE, and CHASE_DB1 datasets, and the results are summarized in Table 6. Each experiment isolates the effect of a specific module by incrementally incorporating them into the base model and observing their influence on key performance metrics.

**Table 6:** Ablation study of the proposed model.

Model Configuration	$A_{cc}$	*SE*	*SP*	$F_{1}$ score	AUC
Base U-Net	0.8540	0.8120	0.8900	0.8400	0.9100
+ Texture branch	0.8760	0.8350	0.9030	0.8560	0.9270
+ Cascade Dilated Convolution Module (CDCM)	0.8930	0.8480	0.9200	0.8670	0.9350
+ Boosting algorithm	0.9100	0.8620	0.9350	0.8760	0.9480
+ Probability Distribution Attention (PDA)	0.9240	0.8790	0.9450	0.8890	0.9580
**Default settings**	**0.9380**	**0.8910**	**0.9600**	**0.9050**	**0.9711**

#### Impact of each component

- *Base U-Net:* The baseline model, without any additional modules, achieves an accuracy of 85.4% and a sensitivity of 81.2%, serving as the foundational structure for retinal vessel segmentation. While it provides a reasonable segmentation, it struggles with capturing fine vessel details, especially in thin and low-contrast regions, leading to relatively lower sensitivity.
- *Texture Branch:* The addition of the texture branch improves both accuracy (87.6%) and sensitivity (83.5%), demonstrating its effectiveness in capturing fine-grained vascular details. The improved performance suggests that integrating textural information helps in better differentiating vessels from the background, particularly enhancing segmentation in areas where intensity variations are minimal.
- *Cascaded Dilated Convolution Module (+CDCM):* Incorporating CDCM leads to a further boost in performance, reaching an accuracy of 89.3% and sensitivity of 84.8%. The ability of CDCM to extract multi-scale features enhances segmentation across vessels of different sizes, making the model more robust to thicker and thinner vessel variations. This highlights the importance of multi-scale context aggregation in retinal vessel segmentation.
- *Boosting Algorithm (+Boosting algorithm):* The inclusion of a boosting algorithm strengthens the model’s ability to refine vessel segmentation predictions, achieving an accuracy of 91.0% and sensitivity of 86.2%. The results indicate that boosting helps reduce misclassifications, particularly in challenging regions where vessel boundaries are difficult to delineate. This demonstrates that iterative learning and refining predictions at multiple levels improve segmentation robustness.
- *Probability Distribution Attention (+PDA):* The integration of PDA significantly enhances performance, improving accuracy to 92.4% and sensitivity to 87.9%. PDA’s role in adjusting feature distributions ensures that both shallow and deep features contribute effectively, which is particularly beneficial in low-contrast regions. The improvement suggests that PDA enhances the model’s ability to distinguish between vessels and background noise, reducing segmentation errors.
- *Full Model (Default settings):* Finally, the complete model, incorporating all proposed components, achieves the highest segmentation performance, with an accuracy of 93.8%, sensitivity of 89.1%, and an AUC of 0.9711. The results highlight the complementary nature of the structural branch, texture branch, CDCM, boosting algorithm, and PDA, demonstrating that each module contributes uniquely to the segmentation pipeline.

This expanded ablation analysis underscores the incremental improvements introduced by each component, providing a clearer understanding of their isolated effects on segmentation performance. The results confirm that while each module enhances specific aspects of vessel segmentation, their combined effect leads to state-of-the-art segmentation performance.

## Conclusion

In this paper, we addressed the limitations of existing RVS algorithms, which often fail to accurately segment the ends of fine vessels and are adversely affected by optical imaging variations and pathological regions. To overcome these challenges, we proposed a novel segmentation method that integrates attention mechanisms and dialated convoluation module offering a more robust and precise solution. Our approach introduces a dual-path U-Net designed to capture both coarse and fine-grained vessel structures through distinct texture and structural branches. By incorporating a CDC module, the model effectively captures multi-scale vessel features, enhancing its ability to extract deep semantic information. This feature extraction capability is crucial for accurately identifying intricate vessel structures across varying scales. Additionally, we employed a boosting algorithm that integrates probabilistic attention distributions within the upscaling blocks. This strategy dynamically adjusts the probability distribution to emphasize shallow features, improving segmentation performance in complex imaging backgrounds and reducing the risk of overfitting. The final output from the dual-path U-Net is then refined through a dedicated feature refinement module, ensuring the integration of relevant features and further enhancing segmentation precision.

Experimental evaluations on three widely used datasets, i.e., CHASEDB1, DRIVE, and STARE demonstrated that the proposed method outperforms existing approaches, delivering superior segmentation accuracy. These results confirm the effectiveness of our method in handling the complexities associated with RVS. The integration of attention mechanisms, multi-path architecture, and feature refinement modules contributes to a more accurate and reliable segmentation process, paving the way for improved diagnostic applications in retinal imaging.

## Data Availability

The source code is freely accessible at https://github.com/Ruihong008/RVS/.
